# Nonlinear Optical Microscopy: From Fundamentals to Applications in Live Bioimaging

**DOI:** 10.3389/fbioe.2020.585363

**Published:** 2020-10-09

**Authors:** Valentina Parodi, Emanuela Jacchetti, Roberto Osellame, Giulio Cerullo, Dario Polli, Manuela Teresa Raimondi

**Affiliations:** ^1^Department of Chemistry, Materials and Chemical Engineering “G. Natta”, Politecnico di Milano, Milan, Italy; ^2^Istituto di Fotonica e Nanotecnologie (IFN) – CNR, Milan, Italy; ^3^Department of Physics, Politecnico di Milano, Milan, Italy

**Keywords:** nonlinear microscopy, stem cells, tissue engineering, label-free microscopy, live imaging

## Abstract

A recent challenge in the field of bioimaging is to image vital, thick, and complex tissues in real time and in non-invasive mode. Among the different tools available for diagnostics, nonlinear optical (NLO) multi-photon microscopy allows label-free non-destructive investigation of physio-pathological processes in live samples at sub-cellular spatial resolution, enabling to study the mechanisms underlying several cellular functions. In this review, we discuss the fundamentals of NLO microscopy and the techniques suitable for biological applications, such as two-photon excited fluorescence (TPEF), second and third harmonic generation (SHG-THG), and coherent Raman scattering (CRS). In addition, we present a few of the most recent examples of NLO imaging employed as a label-free diagnostic instrument to functionally monitor *in vitro* and *in vivo* vital biological specimens in their unperturbed state, highlighting the technological advantages of multi-modal, multi-photon NLO microscopy and the outstanding challenges in biomedical engineering applications.

## Introduction

One of the most fascinating advancements in bioengineering is the possibility to observe and control the microscopic universe of cells in order to understand the biological mechanisms involved in physiological and pathological phenomena of life (Vo-Dinh, 2003). Since researchers are concerned to find high speed, effective, and non-invasive diagnostic tools to identify the state of biological samples, several micro-scale imaging techniques have been developed recently, aiming at applications usable by clinicians on human patients (König et al., 2019). The power of optical microscopy is to provide a non-invasive morphological and functional characterization of the observed living specimen over time (Stephens, 2003). Among the existing microscopy techniques, brightfield microscopy is suitable to observe unlabelled vital and thin samples, and it is the easiest to use. However, monolayered cells appears transparent and contrast methods, like phase contrast microscopy, differential interference contrast microscopy or digital holography, are necessary to distinguish cells or parts of them (Murphy, 2001). Nevertheless, these methods provide low amount of information from live samples, being limited to qualitative estimation on cell morphology and growth (e.g., % area occupied by the cells over time in the field of view) and being capable of simply distinguishing endoplasmic reticulum, nucleus and vesicles. To gain more details, biological specimens can be characterized by means of cyto-histopathological assays, which however, require fixative procedure to color specific cellular structures, thus affecting vitality. Another common technique to enrich the information content achievable from a vital biological sample is fluorescence microscopy (Combs and Shroff, 2017). In this technique, highly specific light-emitting probes (dye molecules, semiconductor nanoparticles, or fluorescent proteins) chemically bound to specific biological targets (i.e., DNA, phospholipids, and proteins) are administered to the live or fixed samples. By exciting the fluorescent species with a lamp or a laser, it is possible to filter the longer wavelength (Stokes shifted) emitted signal, detecting it in either a widefield or a point scanning approach and reconstructing images (Murphy, 2001; Lichtman and Conchello, 2005). Technological advances consist in the addition of automated scanners, vertical stages, high numerical aperture objectives (oil or water immersion), and corrective pinholes and filters to detect fluorescence over the volume of the specimen (<100 μm thick) (Combs and Shroff, 2017). Furthermore, systems equipped with incubator chambers allow performing live imaging and time-lapse microscopy. However, fluorescence-based techniques show limits related to the required short excitation wavelengths with continuous lasers that cause photobleaching of fluorophores, scattering and absorption affecting the signal collection and the penetration depth in thick samples. Moreover, the use of fluorescent probes can influence the vitality of biological specimens and it often involves complex preparatory treatments to ensure a proper binding with their biological target, thus resulting toxic over time (Liu et al., 1999). The advent of nonlinear optical (NLO) microscopy techniques allowed to overcome these limitations exploiting multi-photon processes stimulated by pulsed lasers with infrared wavelengths. NLO microscopy offers deep tissue penetration (>500 μm) since the infrared excitation wavelengths inherently provide a reduced light scattering and absorption (Konig, 2000; Sanderson et al., 2016). Furthermore, NLO processes based on multi-photon excitation overcome the use of staining, allowing one to obtain rich morphological/structural/molecular information from a sample, which shows nonlinear properties and/or distinctive chemical composition. NLO-based microscopy offers inherent advantages with respect to single-photon fluorescence (SPF), such as label-free observations, 3D-sectioning capability, small focal volume and greater penetration depth, thus making functional imaging possible (Helmchen and Denk, 2005; Hoover and Squier, 2013). Despite NLO microscopy comprises several techniques, the most relevant for biological investigations are: two-photon excited fluorescence (TPEF) (W Denk et al., 1990), second and third harmonic generation (SHG and THG) (Barad et al., 1997; P. J. Campagnola et al., 2001) and coherent Raman scattering (CRS) (Evans and Xie, 2008) microscopy.

Two-photon fluorescence allows the visualization of both exogenous (dye molecules, semiconductor quantum dots, and fluorescent proteins such as GFP, RFP, and YFP) (Xu et al., 1996; Drobizhev et al., 2011) and endogenous fluorophores (such as nicotinamide adenine dinucleotide phosphate-NAD(P) H-, flavin adenine dinucleotide - FAD-, flavoprotein -FP-), and it is often exploited in fluorescence lifetime imaging (FLIM) studies, resulting suitable for metabolic and hybrid investigations. SHG microscopy enables the observation of non-centrosymmetric structures in unperturbed biological specimen, for example collagen fibers, myosin filaments and microtubules and their spatial distribution (Campagnola et al., 2001; Chu et al., 2003). THG microscopy is sensitive to refractive index mismatch between structures in the focal plane, allowing to image interfaces for example between aqueous interstitial fluids and lipid-rich structures, such as cellular membranes, lipid droplets and calcified bone (Weigelin et al., 2016). Finally, CRS techniques are based on the coherent excitation of vibrational modes related to specific chemical bonds of the molecules present in the focal volume, such as lipids, proteins, and DNA (Yue et al., 2011; Zumbusch et al., 2013). Coherent anti-Stokes Raman scattering (CARS) and stimulated Raman scattering (SRS) microscopies benefit from the fact that the signal provides information on the chemical composition and the molecular structure, identified in the peaks of the Raman spectrum (Krafft, 2012).

The growing interest in NLO microscopy is testified by the variety of biological studies performed using the previously mentioned techniques in their individual (single-modality), combined (multi-modality) and multi-spectral (multi-color) conditions, resulting in continuously improved acquisition systems. In order to underline the importance of live imaging in the investigation of fundamental biological processes, we provide a background on the physical principles of the different NLO microscopies and we discuss the state of the art and new advanced applications of label-free multi-modal microscopy in living organisms.

## Fundamentals of Nonlinear Optical Microscopy

**FIGURE 1:**
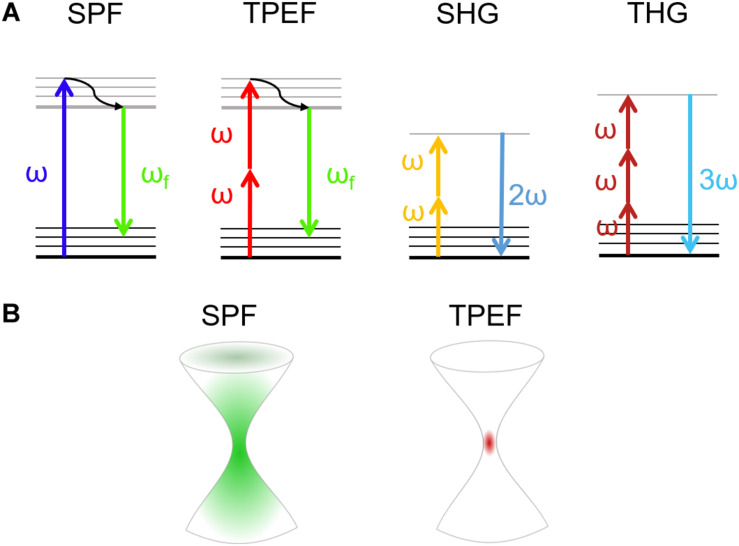
**(A)** Jablonski diagrams involving (from left to right) single-photon (SPF) and two-photon excited (TPEF) fluorescence, and second harmonic (SHG) and third harmonic (THG) generation. **(B)** Size of the excited volume in single-photon (left) and multi-photon (right) fluorescence.

Generally, in SPF microscopy the light sources used to probe the specimen are in the visible wavelength range. As shown in the left side of the panel in Figure 1A, the energy of the incident photon is absorbed by promoting the electron from the ground to the excited state. After non-radiative relaxation in the excited state, the electron returns to the ground state releasing a photon with lower energy (longer wavelength) with respect to the incoming one, thus emitting fluorescence (Williams et al., 1994). On the other hand, when an electron absorbs the energy of multiple photons, it will decay by emitting a photon with higher energy (shorter wavelength) with respect to the excitation photons (Zinselmeyer et al., 2009).

Multi-photon absorption phenomena, exploited in NLO microscopy, are typically achieved with ultrashort-pulsed lasers in the near-infrared wavelength range. NLO processes require a high spatial and temporal density of low-energy photons (Figure 1A; Sanderson et al., 2016). To increase the probability of multi-photon absorption and to gain sufficient signal intensity in NLO microscopy, tightly focused ultrashort pulses are typically used, for which high peak power can be obtained, while keeping the average power comparatively low, thus minimizing the risk of photodamage (Chen et al., 2018).

In NLO microscopy, the strong electric fields achievable in a laser pulse can drive the motion of electrons and atoms to create a polarization which depends nonlinearly on the driving light field, expressed by high-order terms in the electric susceptibility [χ(n) where *n* > 1] (Bloembergen, 1982; Shen, 1985; Di Bartolo and Collins, 2013; Gupta, 2017; Kumar et al., 2018). Due to the strong intensity dependence of the signal, NLO microscopy allows to confine the generation to a very small volume, localized at the laser focus, with respect to the extended excitation obtainable with single photon absorption (Figure 1B). The use of NLO-based techniques is therefore advantageous due to the intrinsic capability of sectioning the specimens. In this way, one can obtain a 3D high-resolution reconstruction of the sample without background noise with just one laser source (Piston et al., 1994).

### Two-Photon Excited Fluorescence

Two-photon fluorescence is a third order NLO process based on the principle of two-photon absorption established in 1931 by Maria Göppert-Mayer in her doctoral thesis (Göppert-Mayer, 1931) and firstly validated 30 years later with the advent of lasers (Kaiser and Garrett, 1961). Here the combined action of two or more photons, which simultaneously interact with the matter, induces an electronic transition from the ground to an excited electronic state. This process is possible when the sum of the energies of these photons is enough to match the energy gap between the fundamental and the excited state. Hence, the most intense excitation is confined within a small volume (voxel) and no out-of-focus light is generated, removing the need for spatial filters (confocal pinholes) to block the unwanted background (Figure 1B; Rubart, 2004). As a matter of fact, since the voxel is small (less than femtoliter for high numerical aperture objectives) and the trajectories of emitted photons are close to the collection angle of objective lens, the TPEF microscopy enables the pulse to penetrate scattering samples in depth, enabling to perform 3D scanning (Denk et al., 1995). Moreover, since the risk of photodamage is limited to the focal volume, TPEF is preferable with respect to single photon fluorescence. The absorption efficiency depends on the physical properties of the fluorophore, the so called two-photon absorption cross-section, and on the excitation light (Helmchen and Denk, 2005; Drobizhev et al., 2011). The unit used to identify this phenomenon is the Göppert-Mayer (GM per photon) that corresponds to 10^–50^ cm^4^ s per photons. Intrinsic molecules such as NAD(P)H are characterized by small cross-sections (<10^–4^ GM), while common fluorescent dyes show absorption cross-sections in the range of 1-300 GM. Hence, the higher is the two-photon absorption cross-section, the higher is the loss in resolution while increasing the peak power (Zipfel et al., 2003a). However, under normal operation the photodamage is very low in TPEF (Hopt and Neher, 2001).

### Harmonic Generation

The harmonic generation is an instantaneous process of coherent nonlinear light scattering, which involves two or three photons in phase matching conditions (Zipfel et al., 2003b). SHG is a second-order NLO process, which happens when two low-energy photons at the same frequency interacting with a nonlinear material are annihilated to generate a new photon with twice the energy of the excitation photons (Campagnola, 2012; Figure 1A). The second-order symmetry of SHG imposes restrictions on active molecular arrays (called “harmonophores”) (Tokarz et al., 2012) and requires the environment to be non-centrosymmetric at the excitation wavelength scale, otherwise the SHG signal vanishes (Campagnola et al., 2001). Since SHG signals occur as a consequence of induced nonlinear polarization instead of absorption phenomena, they exploit the excitation of endogenous factors. The SHG signal has a well-defined polarization, whose anisotropy can be used to determine the hierarchical organization of proteins in tissues. Therefore, the scattering nature of SHG signal drives its intensity emission within a preferential direction. Forward SHG signal is more intense when thin samples are imaged, instead, only backscattered photons can be collected from thick specimens (Mertz and Moreaux, 2001).

In THG microscopy, a third-order NLO process occurs when the fundamental wavelength, typically above 1200 nm, irradiates a medium with intrinsic inhomogeneities due to refractive index mismatch (Yelin and Silberberg, 1999; Rehberg et al., 2011). In THG the combined energy of three photons is converted into a photon emitted at the triple of the energy (a third of the excitation wavelength) (Barad et al., 1997; Figure 1A). In THG microscopy, due to the Guoy phase shift of the fundamental beam, the nonlinear signals generated at symmetric planes with respect to the focus interfere destructively, resulting in zero signal from a homogeneous medium. The THG signal thus reveals the optical heterogeneities of the material illuminated by the laser beam, such as those occurring at the interface between two optically different materials. In contrast to SHG microscopy, no specific symmetries in the material are required to produce a THG signal (Meshulach et al., 1997; Müller et al., 1998).

### Coherent Raman Scattering Microscopy

Raman scattering is a powerful technique for label-free identification of a molecule/material based on the characteristic vibrational spectrum. In spontaneous Raman (SR) microscopy, a monochromatic laser at frequency ω_p_ (“pump”) excites the molecules to a virtual state, which then relax to the ground state scattering photons with lower frequency ω_S_ (“Stokes”). The inelastic frequency shifts Ω = ω_p_ – ω_S_ match the molecular vibrations, which in turn reflect the molecular structure. The resulting SR spectrum provides a detailed picture of the biochemical composition of the measured cells/tissues (Cantarero, 2015). SR microscopy has been translated to clinics e.g., to obtain direct local information from intraoperative analysis of cancer detection (Santos et al., 2016) in human brain (Jermyn et al., 2015; Santos et al., 2017). Despite its advantages, SR microscopy suffers from the drawback of the very weak scattering cross section, due to its incoherent nature, which is 10–12 orders of magnitude lower than fluorescence. This results in low acquisition speed, with pixel dwell times of ≈ 1 s (up to several hours for an image), preventing the acquisition of high spatial resolution images of cells and tissues.

Coherent Raman scattering microscopy overcomes this limitation by generating the Raman signal from a coherent superposition of the molecules in the sample, illuminated by two synchronized ultrashort laser pulses of different color, the pump (at frequency ω_p_) and the Stokes (at frequency ω_S_). When the difference between pump and Stokes frequencies matches a vibrational frequency Ω, i.e., ω_p_ – ω_S_ = Ω, then all the molecules in the focal volume are resonantly excited and vibrate in phase. This vibrational coherence enhances the Raman response by many orders of magnitude with respect to the incoherent SR process, decreasing the acquisition times from seconds to microseconds per pixel. Like other NLO microscopies, CRS provides further advantages: (i) the signal is generated only in the focal volume, allowing three-dimensional imaging; (ii) working out of electronic resonance, it minimizes photo-damage to biological samples.

**FIGURE 2:**
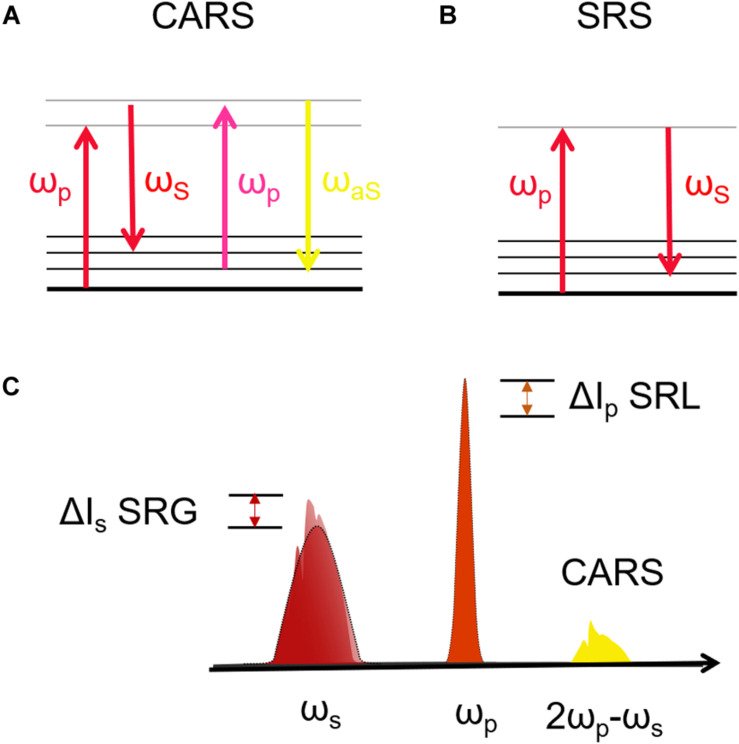
**(A,B)** Jablonski diagrams of CARS and SRS energy transitions. **(C)** Schematic representation of the excitation and emission frequencies involved in SRS and CARS: in evidence the SRS signal in terms of gain (SRG) on the Stokes pulse or the loss (SRL) on the pump pulse.

The two most widely employed CRS techniques are coherent anti-Stokes Raman scattering (CARS) (Cheng and Xie, 2004) and stimulated Raman scattering (SRS) (Nandakumar et al., 2009). In CARS (Figure 2A) the vibrational coherence is read by a further interaction with the pump beam, generating a coherent radiation at the anti-Stokes frequency ω_aS_ = ω_p_ + Ω. In SRS (Figure 2B) the coherent interaction with the sample induces stimulated emission from a virtual state of the sample to the investigated vibrational state, resulting in a Stokes-field amplification (Stimulated Raman Gain, SRG) and in a simultaneous pump-field attenuation (Stimulated Raman Loss, SRL). By comparing CARS and SRS, a delicate balance of advantages and drawbacks emerges: CARS benefits from being a background-free process, since the emitted signal has a frequency ω_aS_ differing from those of pump and Stokes (Figure 2C). In this sense, it is similar to TPEF, SHG, and THG microscopies. On the other hand, it suffers from the so-called non-resonant background (NRB) generated both by the molecular species under study and by the surrounding medium, according to a four-wave mixing scheme. The NRB does not carry any chemically specific information and, when the concentrations of the target molecules are low, can distort and even overwhelm the resonant signal of interest. In addition, the CARS signal scales as *N*^2^, where *N* is the number of oscillators in the focal volume, so that its sensitivity rapidly drops with decreasing oscillator concentration, making it difficult to detect the less abundant biomolecules, and in particular their characteristic signatures in the fingerprint region. The SRS signal is proportional to the imaginary part of the third-order susceptibility tensor χ^(3)^: Since the NRB is a real quantity, SRS is inherently free from NRB. Furthermore, SRS scales linearly with N, thus allowing the detection of weakly concentrated species. On the other hand, SRS requires the detection of a weak differential transmission signal (SRG/SRL) sitting on the large background given by the Stokes (pump) light. Extraction of this signal calls for the use of sophisticated techniques, involving high-speed modulation and lock-in detection, to overcome the laser fluctuations and achieve shot-noise limited detection. Such techniques are particularly challenging to implement at high speeds, with integration times of few tens of microseconds, and over a broad spectrum, required to extract the entire Raman fingerprint of the molecules (Wang et al., 2007; Gupta, 2017; Kumar et al., 2018). Briefly, CARS microscopy can be preferentially performed on high molecular concentration specimens, while SRS with high-sensitive and high-speed acquisition systems can be performed on less chemically dense samples (Kumar et al., 2018).

Table 1 summarizes the physical principles, the main advantages and limitations that still affect the above-described techniques within biophysical application.

**TABLE 1:** Summary of the main properties of the label-free NLO microscopy techniques from physical principles to advantages and limitations.

Tech	Principle	Contrast mechanism	Biological target	Advantages	Limits
TPEF	Two low energy photons simultaneously absorbed to excite a fluorescent species	Fluorescence	Endogenous fluorescent proteins and molecules, cell autofluorescence	• High penetration depth (>500 μm) • High resolution below the diffraction limit of light • High sensitivity, down to the single molecule • Intrinsic 3D scanning capability • Applied *in vitro*, *in vivo* (intravital) and in fixed samples • Can be coupled with FLIM, FRET and FRAP techniques	• Risk of photodamage
SHG	Two photons at the same frequency simultaneously interact with nonlinear species to generate a photon with the double frequency	Second-order nonlinear polarization of non-centrosymmetric structures	Non-centrosymmetric biological structures such as: collagen fibrils I/II, myofibrils, myosin filaments, microtubules	• Intrinsic 3D scanning capability • Fibrillar orientation dependence • Usually coupled with TPEF • Applied *in vitro*, *in vivo* (intravital) and in fixed samples • Low risk of photodamage • Easy interpretability	• Backscattering signal is less intense than forward one. • Fixation did not affect signal generation except in microtubular imaging • Low intensity signal
THG	Three photons simultaneously interact with a medium with refractive index mismatch to generate a photon with the triple frequency	Refractive index mismatch within inhomogeneous medium	Interfaces between lipid and water, cell membranes and vesicles	• Intrinsic 3D scanning capability • Completely label-free • Applied *in vitro*, *in vivo* (intravital) and in fixed samples • Low risk of photodamage	• High infrared wavelength (>1200 nm) • Depend on distribution of signal eliciting-structures and incoming light polarization
CARS	The difference in frequency between the pump and Stokes pulses match the vibrational resonance of the chemical bond emitting an anti-Stokes photon	Anti-Stokes frequency match the resonance frequency of the target molecular bond	Chemical bond resonant frequency from fingerprint region (nuclei acids and proteins) to the C-H stretch region (lipids and proteins) of the Raman shift	• Chemically selective technique • Intrinsic 3D scanning capability • Completely label-free • Applied i*n vitro*, *in vivo* (intravital) and in fixed samples • Multicolor imaging	• Non-resonant background contribution • Phase mismatch • Electronic contribution from background molecules • Four-wave mixing process • Risk of photodamage • Fixation process may perturb the chemical stability of target chemical bonds
SRS	The difference in frequency between the pump and Stokes pulses match the vibrational resonance of the chemical bond enhancing the vibrational transition emitting a photon	SRG or SRL on the Stokes or on the pump pulse, respectively, match the resonant frequency of the target molecular bond	Chemical bond resonant frequency from fingerprint region (nuclei acids and proteins) to the C-H stretch region (lipids and proteins) of the Raman shift	• Chemically selective technique • Intrinsic 3D scanning capability • Completely label-free • Same spectral profile of spontaneous Raman scattering • Fast acquisition process enables real time monitoring of biological processes • Low risk of photodamage • Multicolor imaging • No background fluorescence • Non-resonant background free	• Difficult signal isolation from incoming fields • Fixation process may perturb the chemical stability of target chemical bonds

## Biological Applications

**FIGURE 3:**
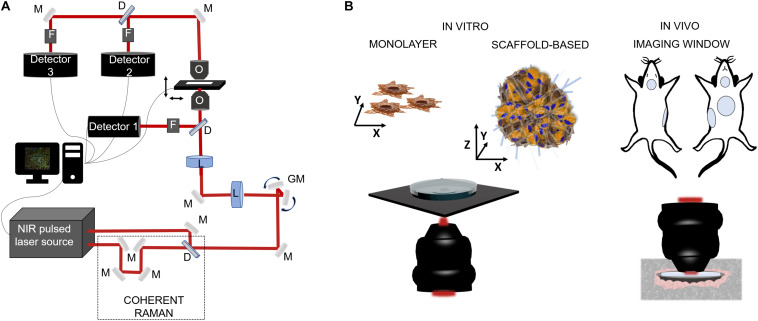
Schematic representations of NLO microscopy application in biology. **(A)** Example of a NLO set-up characterized by a NIR laser source (two in the case of coherent Raman), a set of fixed (M) and mobile (galvanometric mirrors-GM) mirrors and lenses (L) to drive the light toward the focalization objective (O) and then to the sample which is positioned on a motorized stage. The collection of the emitted light can be in reflection by the use of a dichroic mirror (D), a set of filters (F) (to exclude the excitation component from the reflected light) and, a photodetector, or by transmission thanks to a collection objective or a condenser positioned above the sample. **(B)** On the left side, cell model for *in vitro* inspections: single cell analysis on cellular monolayer or 3D cellular constructs composed by heterogenous cell population and a scaffold matrix. The microscope configuration can be in reflection modality, to maintain sample sterility, or in transmission, exposing sample to immersion objective and contaminants. On the right side, intravital microscopy for preclinical analysis is usually made by the use of imaging windows for repetitive observations on the same site for small rodent models. *In vivo* imaging is made in reflection modality using of cover-glasses sealed on the skin of the animal in specific locations, i.e., mammary glands, cranial, abdominal, and tracheal sites.

Nowadays, there is an ever-growing need to develop diagnostic techniques capable to reveal and quantify at the cellular level the expression of pathological processes that affect human health. A valid solution is represented by NLO microscopy techniques which allow to perform label-free, vital and three-dimensional observations in biological specimens, from *in vitro* samples to preclinical *in vivo* models and, potentially, to clinical applications. In the last 30 years, NLO microscopy techniques have been applied to a broad range of biomedical problems, such as tumor infiltration and growth (Lu et al., 2015; Gavgiotaki et al., 2017; De Bortoli et al., 2018; You et al., 2018; Sun et al., 2019), stem cell differentiation and proliferation (Quinn et al., 2013; Meleshina et al., 2016, 2017), tissue regeneration (Yu et al., 2012) and repair (Huemer et al., 2017; Rico-Jimenez et al., 2020). In Figure 3A a schematic representation of a NLO microscope in inverted configuration, in which the excitation light irradiates the sample from the bottom to the top. The collection of the emitted signal can be performed both in reflection than in transmission modality, depending on the sample thickness. In biological applications, several specimens can be imaged, as shown in Figure 3B. As described in the following sections, *in vitro* monolayer cell models are easy to investigate since thick and transparent media facilitates the orientation and the focusing. Within that, the detection of the generated signal can be dual, especially in case of fixed specimens, without taking care of sterility in case of contact with immersion objectives and environmental air. *In vivo* analyses, on the other hand, are more difficult since they can be preferentially performed in reflection. Hence, imaging windows for long-term time-dependent studies are often created in specific sites of the animal, thus resulting highly invasive. For this reason, an intermediate application of NLO microscopy to both replicate the heterogenous *in vivo* environmental conditions without involving direct animal experimentations, is from bioengineering cellular constructs. Thus, 3D, vital and functional imaging of a more realistic patho-physiological condition with respect to cell monolayer and less invasive than preclinical model, is guaranteed.

### Nonlinear Imaging of Cellular Models

**FIGURE 4:**
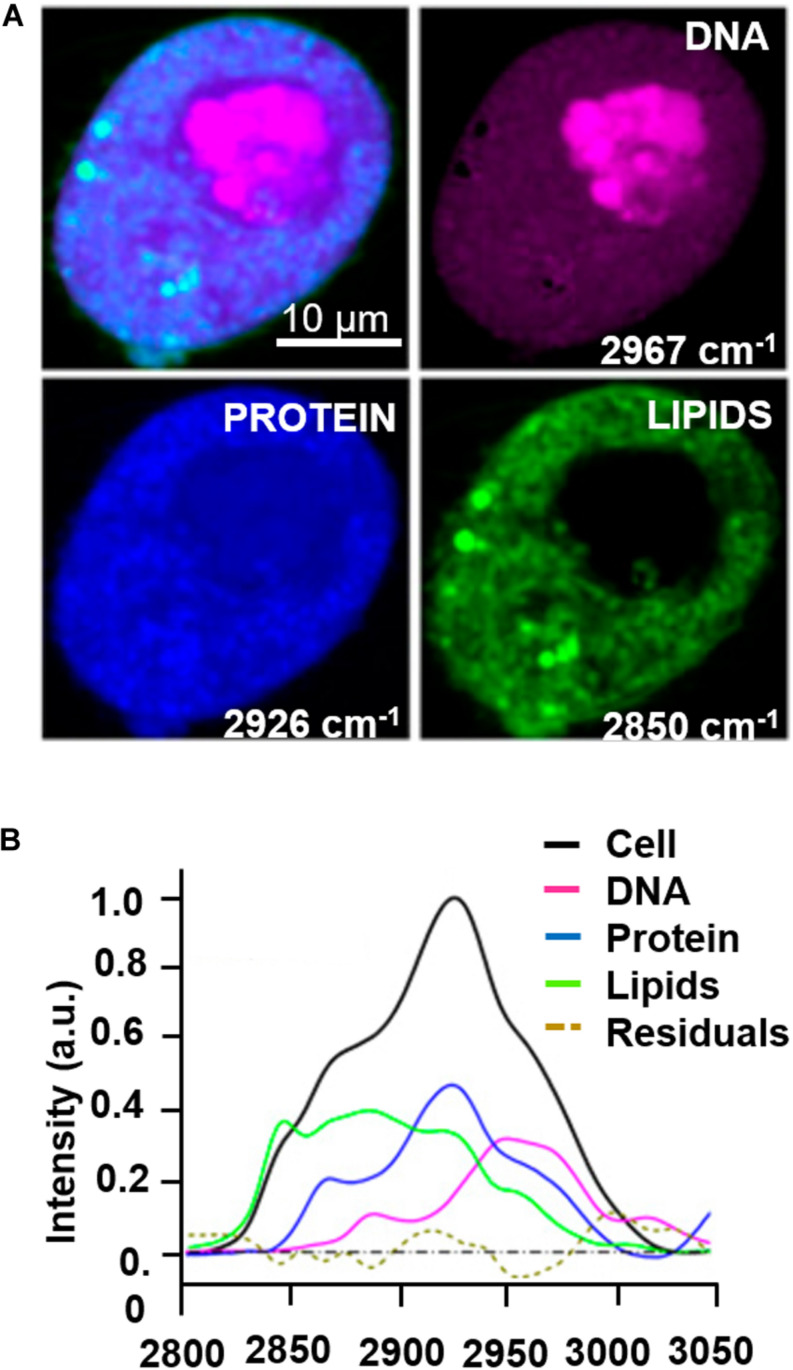
Example of multi-color SRS microscopy on living tumor cells during prophase: **(A)** DNA (magenta) at 2967 cm^–1^, proteins (blue) at 2926 cm^–1^ and lipids (green) at 2850 cm^–1^. **(B)** Raman spectrum extracted from the cell pellet showing the signatures of the different species. Scale bar = 10 μm. Figures adapted from (Lu et al., 2015) for kind permission of PNAS.

The majority of label-free, vital NLO microscopy techniques in biology are applied *in vitro* using cell culture models that allow to reproduce and to control complex phenomena such as lineage differentiation of stem cells (Quinn et al., 2013; Meleshina et al., 2016, 2017), cell response to drugs (Fu et al., 2014; De Bortoli et al., 2018), synthesis or inhibition of single cellular structures (Zhang et al., 2012; Van Steenbergen et al., 2019). Table 2 summarizes a selection of representative biological studies on vital cultured cells through NLO microscopy published during the last decade. Several works involve the use of multi-frequency SRS microscopy, since it is the most recently developed technique able to provide molecular information of less chemically concentrated biological specimens without the signal impairments typical of CARS, i.e., NRB. Moreover, SRS microscopy is frequently applied to the study of tumor microenvironment, cancer cell composition and dynamics (Zhang et al., 2012; Lu et al., 2015) since the outcomes are often comparable with their histopathological counterparts using conventional hematoxylin and eosin staining (Ji et al., 2013; Chan, 2014). Fa-Ke Lu published in 2015 (Lu et al., 2015) an innovative study based on label-free DNA imaging in cancer cells during their mitotic phase by the use of multi-frequency SRS microscopy (Figure 4A). Both the fingerprint region and the C-H-stretch region of the Raman spectrum were investigated. The best result was reached by linear decomposition of three distinctive peaks at different wavenumbers in the C-H-stretching region thus subtracting the strong protein background and enhancing the Raman signal of the distinct cellular structures (lipids, proteins and nucleic acids) (Figure 4B). Another example of NLO imaging in biology is represented by the study of Meleshina et al. (2016, 2017) based on TPEF-FLIM and SHG microscopy. FLIM was employed to evaluate the metabolic rate of human mesenchymal stem cells (MSCs) during controlled differentiation toward adipogenic, chondrogenic and osteogenic phenotypes at different time-points. SHG microscopy, allowed to monitor collagen formation during chondrogenesis. While differentiation occurred, a metabolic shift was estimated from redox ratio of FAD/NAD(P)H and the lifetimes of bound and free state of NADH in all the phenotypes investigated. The application of THG microscopy is testified by the work of Wu et al. in 2016 (Wu et al., 2016) since different type of white blood cells from the immune system showed distinctive morphological features and intrinsic organelles distribution. Similarly, Gavgiotaki et al. (2017) demonstrated that a discrimination among several sub-types of breast cancer cells was possible by the use of TPEF and THG imaging comparing primary, immortalized cells with peripheral mononuclear cells (control). CARS microscopy is a powerful technique to follow lipid production because the large number of C-H bonds in lipid molecular tail corresponds to high Raman signal intensity (Cheng and Xie, 2004). Saarinen et al. (2017) applied CARS microscopy to a transwell culture configuration on an intestinal epithelium model of Caco-2 cells to evaluate drug permeability throughout the cell layer over 21 days of culture monitoring lipid formation. Another interesting study at the cell scale is from Van Steenbergen et al. (2019) where SHG microscopy is used to observe microtubular structures, a cytoskeletal component involved in protein intracellular transport and mitosis, in neural cells axons derived from different regions of the nervous system. This unusual application of SHG was aimed to validate label-free microscopy as a promising alternative to immunofluorescence staining, since microtubules can be imaged in their native and dynamic configuration. Moreover, this group demonstrated that cell fixation and dehydration with acetone, methanol and common aldehydes highly impaired SHG emission in microtubules, suggesting the potentialities of this technique for imaging untreated live cells. Although Van Steenberg et al. demonstrated that SGH is suitable for investigating in label-free condition single cell process, limitations due to the weakness of the generated signal and the microtubular concentration-dependence of the cell type need to be overcome.

**TABLE 2:** Most recent *in vitro* studies where NLO imaging had been applied in vital and label-free conditions.

References	Tech	Cell type	Study	Main findings and criticalities
Zhang et al. (2012)	SRS	Salivary gland cells of drosophila melanogaster and human epithelial cell line (HEK-293) and human breast cancer cell line (MCF-7)	*In vitro* study of the distribution of nuclei acids, proteins, and lipids with fingerprint SRS of salivary gland cells of *drosophila larvae* and mammalian cells	Assignment of single Raman resonant peaks corresponding to the single biochemical components. Drosophila cells allowed to imaging high level of DNA content with SRS thus tailoring the system for imaging mammalian cells
Quinn et al. (2013)	TPEF	Human mesenchymal stem cells (MSCs)	Evaluation of the metabolic changes associated with *in vitro* human mesenchymal stem cell differentiation via NADH and FAD autofluorescence and their relative redox state FAD/(NADH+FAD)	Correlation between the optical redox ratio and concentration of cofactors estimated by conventional approach (liquid chromatography); overall decrease in redox ratio describes the beginning of differentiation and a new synthesis of fatty acids within adipogenic differentiation. During differentiation process a clustering of mitochondria was found out
Fu et al. (2013, 2014)	SRS	Murine bone marrow-derived cell line (BaF3)	*In vitro* temporal visualization and quantification of tyrosine kinase inhibitor, a drug for the treatment of chronic *myelogenous leukemia* in cancer cells	Imaging of drugs accumulation within the cells was independent from the binding to the relative target: SRS spectra of investigated drugs resembled the spontaneous one. SRS reveal lysosomal enrichment over time of about 1000-fold of drugs after few hours and the interaction of chloroquine with tyrosine kinase showed a reduction of imatinib and an enrichment of nilotinib in the lysosome
Lu et al. (2015)	SRS	Human cancerous cervical cell line (HelaS3)	*In vitro* and *in vivo* study of chromosome dynamics inside nuclei during mitosis, *in vivo* cell proliferation in mouse skin induced by drug treatment and label-free histology method for human skin cancer diagnosis through SRS	Linear decomposition reveals three distinctive resonance peaks in the C-H stretch region for DNA, lipids and proteins. Imaging of cell mitosis at different time points: 0, 10, 20, and 30 min
Pope et al. (2013); Bradley et al. (2016)	TPEF CARS	Mouse oocytes and embryos	*In vitro* and *in vivo* label-free study of the number, size and distribution of lipid droplets in mouse oocytes and embryos	Quantification of lipids at different levels of Z-stack images of the same embryo. Estimation of maturation capacity of eggs after CARS imaging. Comparison between CARS of lipids and fluorescence staining (BODIPY) on fixed eggs (co-localization verified). Hyperspectral CARS imaging of eggs, oocytes and embryos to obtain a developmental trend of chemical components
Meleshina et al. (2016, 2017)	TPEF (FLIM) SHG	Human MSCs	Non-invasive study of *in vitro* mesenchymal stem cell differentiation toward adipogenic, osteogenic and chondrogenic phenotype through metabolic variation of NAD(P)H, FAD and SHG to assess collagen presence	Redox ratio and lifetime of bound NAD(P)H showed a general metabolic shift toward glycolysis during differentiation. Within this osteogenic differentiation was preceded by an oxidative phosphorylation
Wu et al. (2016)	THG	Human leukocytes	A novel approach applied to cytometry: discrete analysis of circulating and/or suspended cells from the immune system both *in vitro* and *in vivo* on human patients through the skin	Isolation (extraction and separation with cell sorter) and observation with THG of white blood cells avoiding environmental stresses and changes in cell morphology were done before and after cytometry. Differentiable features to distinguish cell type were THG intensity and cell size after image segmentation. Neutrophils, monocytes and lymphocytes have been identified from different donors
Gavgiotaki et al. (2017)	TPEF THG	Human breast cancer cell lines (MCF7, BT474, MDA-MB231), primary non-tumorigenic human breast cancer cells (MCF10a) and human peripheral blood mononuclear cells	Validation of THG technique to diagnose and distinguish different subtypes of breast cancer cells through qualitative and quantitative analyses compared to control cells (peripheral mononuclear blood cells)	Distinction of THG signaling pattern of different cell types and subtypes in terms of cellular and intracellular membranes. Co-localization of Nile red staining identified with TPEF and THG of intracellular lipid vesicles. THG signal intensity and distribution from nuclei, cellular membranes and intracellular membranes allowed to distinguish cell types compared with FTIR analysis
Saarinen et al. (2017)	CARS	Intestinal epithelial cell line (Caco-2)	Evaluation of drug permeability of Caco-2 cells grown in transwell membrane inserts by lipid droplets detection	Membrane inserts of transwell culture plates made by PTFE, PET and PC were tested: PTFE resulted more durable during CARS. Cell viability after CARS imaging on Caco-2 showed photodamage (morphological variations and cell detachment) increasing laser powers over 40 mW (pump) and 20 mW (Stokes). CARS at 2840 cm^–1^ showed lipid formation after 7 days of culture and lipid droplets growth in size over 21 days
Crisafi et al. (2018); De Bortoli et al. (2018)	CARS	MDA-MB-231 and MDA-MB-157 human breast cancer cell lines	Study of the chemical composition of intracellular vesicles *in vivo* and *in vitro* model of human breast cancer cells after iron depletion	CARS allowed to identify the chemical composition of vacuoles produced by cancer cells after iron depletion, mainly composed of medium surrounded by lipid shells
Sun et al. (2019)	TPEF THG	Mouse melanoma cell line (B16-F10)	Comparative study of melanin deposition with different concentrations in live melanoma cell line model via THG microscopy. TPEF detected the signal intensity as the indicator of the melanin concentration	Comparison between synthetic and natural melanin via THG and TPEF mean intensity quantification respect to melanin mass density
Van Steenbergen et al. (2019)	SHG	Primary mouse neuronal cells (hippocampal, enteric nervous system, dorsal root ganglion cultures)	Axonal microtubule network and microtubule polymerization/depolymerization (colchicine and nocodazole treatment) in neurodegenerative pathologies. Deepening of the knowledge related to SHG molecular detection in weak harmonophores	SHG signal originated from guanosine-5’-triphosphate (GTP)-bound tubulin dimer conformation in the plus-end and along the microtubule network. SHG signal generation depended on microtubules polarity, number and organization (constructive interference). Post-treatment with Taxol, increase SHG signal from more stable GTP-bound tubulin in less organized microtubular configurations in another cell type apart from neuronal cells. Fixation with acetone, methanol and paraformaldehyde alters the microtubules SHG signal

### Label-Free Nonlinear Microscopy in Animal Studies

**FIGURE 5:**
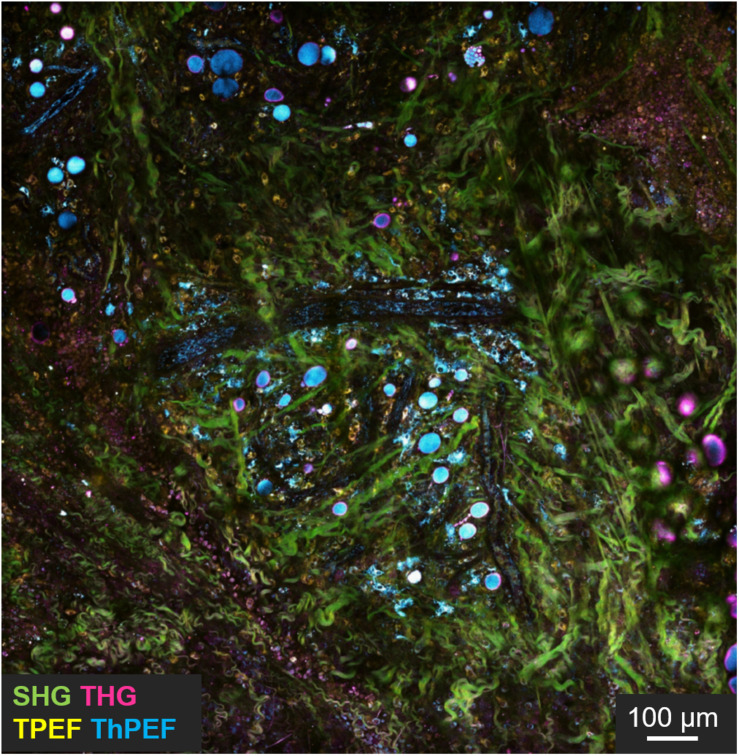
Intravital imaging of mouse tumor microenvironment showing SHG (green), THG (magenta), TPEF (yellow), and three photon fluorescence (cyan) signals. In evidence the green signal from collagen fibers, cyan from NADH, yellow from FAD and magenta from interfaces. Scale bar = 100 μm. Figure reproduced from You et al. (2018) with kind permission from Springer-Nature Communications and licensed by s100.copyright. com\AppDispatchServlet.

Completely label-free and vital NLO microscopy applications *in vivo* are less available in literature since these acquisition techniques are often hybridized with fluorescent tags *in situ* or directly *ex vivo* on freshly/cryopreserved tissue slices to improve the orientation within heterogenous samples. Adapting the design of a microscope for animal or human imaging often requires reflection configuration (epi-detection) coupled with immersion objectives and fixative stages to reduce image blurring, aberrations and resolution losses. Table 3 collects *in vivo* applications of NLO microscopy on vital and unlabeled animal models. Imaging small organism such as zebrafishes (Yu et al., 2012; Huemer et al., 2017), is relatively common in developmental and regenerative time-lapse microscopy thanks to the small size and the low cost related to animal growth. However, the mouse model is still the most used animal in biology to resemble human physiopathology. In NLO microscopy, this is especially employed to reproduce the tumor infiltration dynamics. In fact, You et al. (2018) presented an innovative NLO multi-modal imaging system based on four photodetectors to speed up the acquisition time by sequentially collecting autofluorescence signals of FAD with TPEF, NAD(P)H with three-photon fluorescence, extracellular matrix (ECM) with SHG, and interfaces through THG microscopy on intravital mouse model of mammary gland tumor (Figure 5). Despite the high quality and the good level of interpretability of the results, the intravital approach is highly invasive since a direct access to the imaging site is necessary. The animal underwent surgery to expose the tumor mass and at the end it was euthanized. Similarly, Alonzo et al. (2016) performed a metabolic study by TPEF microscopy coupled with time-resolved analysis of NAD(P)H and FAD of adipose cells from white, brown and induced-beige adipose tissue, to assess the thermoregulatory property of fat. *In vivo* observations confirmed the estimations previously obtained *ex vivo*: time-variant metabolic profiles corresponded to a different function of tissue, thus offering more significant results with respect to gene expression analysis. Instead, Lu et al. (2015) studied the proliferation kinetics of tumor cells via multi-color SRS microscopy through a dorsal skinfold chamber. This system is based on a thin layer of skin stretched and encaged between two glasses, thus creating a window that allows optical accessibility to monitor tissue growth over time, and minimizing animal movement and ensuring repetitive analysis in the same area of the sample. A time-dependent analysis of scar tissue formation after superficial dorsal wounding, has been presented by Rico-Jimenez et al. (2020). A comparative preclinical analysis of diabetic mouse model exposed to placebo, low and high dose of drug (hypoxia-inducible factor coupled with prolyl hydroxylase inhibitor) allowed to reveal, via TPEF-FLIM and SHG microscopy, wound healing, angiogenesis, and metabolic behavior differences among cell populations in the injured site at different time points. In this work, the animal survival was guaranteed for the whole duration of the experiments, allowing repetitive observations of the same area without invasive surgical intervention and imaging window implant.

**TABLE 3:** Intravital and *in vivo* NLO imaging in label-free and vital conditions.

References	Tech	Model	Study	Level of invasiveness	Main findings and criticalities
Yu et al. (2012)	CARS SRS	Cephalochordate amphioxus and zebrafish	*In vivo* study of the chemical composition of the notochord structure in *cephalochordate amphioxus* and zebrafish through non-invasive CARS and SRS microscopy	Low	Large SRS images of notochord of both lipids (2845 cm^–1^) and proteins (2950cm^–1^). Compared to CARS, SRS did not suffer from non-resonant background thus resulting clearer in protein visualization
Lu et al. (2015)	SRS	Mouse	*In vitro* and *in vivo* study of chromosome dynamics inside nuclei during mitosis, *in vivo* cancer cell proliferation in mouse skin (dorsal skinfold chamber) induced by drug treatment and label-free histology method for human skin cancer diagnosis through SRS	Medium	Observation of cell division on mouse skin after epidermal carcinogenesis induction through a dorsal skinfold chamber: strong DNA signal with well-defined condensed chromosomal components at different time-points. Lipids, proteins and nuclei resulted well-defined and visible by linear decomposition of resonant frequencies in the C-H stretch region
Alonzo et al. (2016)	TPEF (FLIM)	Mouse	3D functional imaging of different types of adipocytes related to their metabolic activity in terms of fluorescence lifetime of NADH and FAD and their relative redox ratio. The thermogenic function of the adipose tissue corresponded to a different metabolic activity	High	Functional metabolic imaging enabled to distinguish thermogenic from non-thermogenic adipose tissue. White and brown adipose tissue had different lifetimes comparing *ex vivo* tissue slices with vital mouse
Huemer et al. (2017)	SHG	Zebrafish	*In vivo* long-term time-lapse and 3D imaging of wound healing on zebrafish using an engineered entrapment device for high-resolution microscopy	High	Design of specific entrapment cage for optical study of larvae tail regeneration. SHG compared to fluorescence confocal microscopy give more defined and label-free results at different time points for time lapse observations
Wu et al. (2016)	THG	Human	A novel approach applied to cytometry: discrete analysis of circulating and/or suspended cells from the immune system both *in vitro* and *in vivo* on human patients through the skin	Low	Identification of white blood cells circulating in the dermal papilla region beneath the skin via video-rate THG. Cell types were distinguished by large cross-sectional area, smaller size and area. Preliminary clinical experiment
You et al. (2018)	TPEF ThPF SHG THG	Mouse	Simultaneous label-free and time lapse microscopy of FAD and NADH autofluorescence (2 and 3 photon fluorescence) and SHG/THG signal to track cell interaction within extracellular components *in vitro* and i*n vivo* mouse mammary gland tumor model	High	Observation of tumor microenvironment in live animal model at different depths. Live observation of leukocyte recruitment in the circulatory system as indicator of innate immune response. Different phases of this process had been identified since a reduced velocity of blood cells was evident. Leukocytes were followed in their trans-migratory and interstitial nature in the pathological site
Rico-Jimenez et al. (2020)	TPEF (FLIM) SHG	Mouse	Study of the wound healing process and scar tissue formation in diabetic mice (four groups: control, placebo, low and high concentration of drug) to investigate the metabolic changes that occurs in cell populations of the skin and the signaling cascade involving angiogenesis and collagen fibers organization	Medium	Longitudinal tracking of wound healing and quantification of physiological changes during healing. Pharmacodynamic prediction based on imaging results within the repair mechanism was provided
					

Nowadays, although the NLO techniques presented have allowed a wide range of label-free observations with increasingly high-performance, they are still limited in transferring their application from the bi-dimensional *in vitro* model to complex *in vivo* samples. Hence, the big challenge of label-free imaging of vital animal models remains to be addressed due to the high level of difficulty when observing several tissue layers and multiple packed populations of cells. A step in between, would be to characterize bioengineered cellular models aimed at emulating *in vitro* the biological environment in its structural, physical and chemical compositions for modeling tissue regeneration or for local tissue repair. Therefore, the advantages offered by the imaging of *in vitro* systems are combined with the study of systems near to the preclinical model without incurring the costs and limits of animal experimentation and allowing to obtain equally useful data for scientific research.

In the following section, we will present exemplificative applications of nonlinear imaging in tissue engineering and our preliminary steps in multi-modal NLO microscopy based on a 3D synthetic scaffold for cell culture.

## Nonlinear Microscopy Toward 3D Bioengineered Systems

**FIGURE 6:**
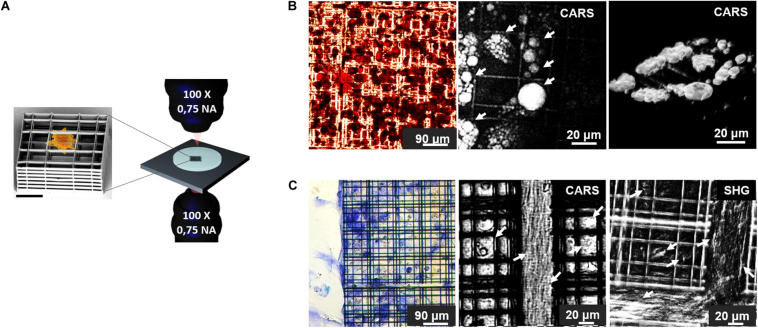
**(A)** Schematic representation of the experimental model employed in this work: on the left, SEM image of a single element of Nichoid 3D synthetic scaffold (Scale bar = 20 μm) used for MSCs culture. Nichoids design was made of three lattice parallel grids with graded pore size and an overall thickness of 30 μm to enable optical accessibility. On the right a rendering of the transmission inverted NLO microscope for multi-modal TPEF, SHG, CARS, and SRS microscopy. **(B)** Adipogenic differentiation of MSCs after 2 weeks of culture. On the left, oil red-O colorimetric assay of fixed cells, showed lipid droplets formation in red, while in the center, CARS image, of the same sample acquired before fixation and oil red-O staining, showing lipid droplets, resonant at 2845 cm^–1^ and indicated by white arrows. On the right a 3D rendering of CARS signal acquired by sequential imaging at variable depth inside the sample and stacking the images. **(C)** Chondrogenic MSCs differentiation after 3 weeks of culture. On the left, toluidine blue assays performed on fixed specimen, revealed production of acidic component of ECM stained in blue. In the center, CARS imaging at 2940 cm^–1^ Raman shift, resonant with collagen (highlighted by arrows), in untreated, live, and differentiated sample. On the right side, SHG image acquired on vital stem cells after 21 days of differentiation, white arrows indicate collagen fibrils. Autofluorescence signal from the Nichoids material is present and more evident in SHG modality.

In the last decade, it has been demonstrated that non-invasive nonlinear microscopy represents a promising strategy for label-free live imaging of 3D engineered tissues. In fact, several studies had been performed to characterize morphology (Hofemeier et al., 2016; Nguyen et al., 2017; Syverud et al., 2017; Costa Moura et al., 2018; Kaushik et al., 2019; Moura et al., 2019), functionality (Hofemeier et al., 2016; Li et al., 2017; Syverud et al., 2017; Cong et al., 2019; Okkelman et al., 2020), composition and distribution of chemicals (Hofemeier et al., 2016; Li et al., 2017; Syverud et al., 2017; Costa Moura et al., 2018; Moura et al., 2019; Sood et al., 2019), invasion, infiltration and mechano-regulation of cellular constructs (Hofemeier et al., 2016; Nguyen et al., 2017; Syverud et al., 2017; Costa Moura et al., 2018; Kaushik et al., 2019; Moura et al., 2019; Sood et al., 2019), which have been collected in Table 4. A representative study made by Hofemeier et al. (2016) carried out a multi-spectral CARS and SHG imaging on an engineered bone tissue from stem cell differentiation toward osteogenic phenotype within 3 weeks of culture. Similarly, Syverud et al. (2017) generated a fully-differentiated musculoskeletal tissue and exploited NLO microscopy with TPEF for imaging the autofluorescence of NAD(P)H, and SHG to identify collagen and myosin fibers. This work established label-free NLO microscopy as a valid alternative to collagen and myosin immunostaining, even if the tissue construct was fixed and sliced before the acquisitions, and only monolayered cells were imaged in their entirety and vitality. Contrarily, Moura et al. (Costa Moura et al., 2018; Moura et al., 2019) investigated the composition and the distribution of collagen, proteins and lipids with bioengineered cartilage constructs by the use of multi-modal TPEF, SHG and CARS microscopy both in the fingerprint than in the C-H stretch region of the Raman spectrum. To date, Moura provided a comparison between SHG and CARS imaging of collagen to highlight the difference in signal distribution and observed the synthetic constructs in their thickness. Another innovative example of label-free, vital NLO imaging in tissue engineering is the study of Kaushik et al. (2019) which observed blood vessels formation in a dynamic bioreactor providing multi-well plates with media-flow re-circulation circuits. By monitoring the endogenous TPEF from the cells encapsulated and differentiated within a 3D hydrogel, they were able to reconstruct the network in its three-dimensionality. Furthermore, diameter, density, branch nodes amount and anisotropy of self-assembled blood vessel networks in static, recirculating and continuous flow conditions were estimated from image post-processing. Unfortunately, this study was limited by the duration of the acquisition which prevented the analysis over multiple days. The biological investigations described previously, performed on vital adherent and suspended cells, spheroids/organoids, cellular constructs and small-sized animals, exclusively by the use of label-free NLO microscopy, provided functional information otherwise difficult to obtain through conventional methods. Limited versatility of the acquisition systems, poor optical accessibility of the engineered tissue construct and slow acquisition speed may require further technological improvements in this field. In our group, we built a customize transmission inverted multi-photon NLO microscope designed to perform parallel CARS and SRS microscopy (Crisafi et al., 2017, 2018) and recently adapted also for SHG and TPEF microscopy with pulses < 100 fs. The advantage offered from our personalized acquisition system relies in the possibility to perform in series and on the same area of a sample, CARS, TPEF, SHG, and SRS imaging by filtering properly the emitted signals. We applied these technologies on an optically accessible *in vitro* experimental model to investigate the behavior of rat bone marrow-derived MSCs growth in a microfabricated 3D scaffold, named Nichoid (Nava et al., 2012; Raimondi et al., 2013; Zandrini et al., 2019) with the aim to perform serial 3D scanning multi-modal NLO imaging over time (Figure 6A). For example, MSCs were cultured inside Nichoids and differentiated toward adipogenic and chondrogenic phenotypes and they were characterized with both conventional biochemical assays and NLO microscopy. Conventional results on the cellular adipogenic phenotype were obtained from bright field imaging of cyto-histological oil red-O staining of lipid droplets produced inside cells after cell fixation and staining. This technique is effective and fast but, unfortunately, returns only qualitative results. On the other hand, CARS microscopy provided the chemical selectivity for lipids, resonant at 2845 cm^–1^ Raman shift, in vital and unperturbed adipocytes, also allowing to distinguish the droplets volumetric distribution with higher spatial resolution (less than 1 μm of axial resolution) (Figure 6B). Similarly, conventional approach to recognize chondrogenic phenotype, revealed the ECM formation within Nichoids after cell fixation and staining with toluidine blue staining. Conversely, SHG and CARS imaging (2940 cm^–1^) enable to observe the distribution and quantify the level of collagen, in vital cultured MSC growth inside Nichoids (Figure 6C). In conclusion, our multi-modal set-up, just switching among different techniques (TPEF, SHG, and multi-color CARS/SRS) is able to perform functional imaging on several tissue and on optically accessible 3D device to investigate the phenomena governing the healthy and pathological conditions of cells.

**TABLE 4:** Examples of NLO microscopy techniques applied to 3D bioengineered systems in vital and label-free conditions.

References	Tech	Cell type	Study	Main findings and criticalities
Hofemeier et al. (2016)	SHG CARS	Human inferior turbinate stem cells	Study of markers of *in vitro* induced osteogenesis of stem cells by hydroxyapatite calcium deposit detection through CARS and SHG at different time points (every 7 days for 3 weeks)	Multimodal imaging to follow the osteogenic differentiation of the cell construct reveal calcium deposits and collagen formation with CARS (959 cm^–1^) and SHG from 14 days to 21 days after the beginning of the differentiation respect to undifferentiated samples
Fu et al. (2013), Li et al. (2017)	SRS	Human ovarian cancer cell line (COV362 and OVCAR5) and primary human ovarian cancer cells	*In vitro* single ovarian cancer cell analysis through hyperspectral SRS to assess the level of saturation of intracellular lipids both in 2D and in 3D spheroids	Study of lipid composition inside cells from fingerprint to C-H stretch region. Increased level of lipid unsaturation in spheroids respect to monolayer cells. Inhibition of lipid desaturation limits ovarian cancer cell stemness and spheroids growth
Nguyen et al. (2017)	TPEF SHG	Human induced pluripotent stem cells (iPSCs) derived from chondrocytes	3D bioprinted iPSCs within nanofibrillated cellulose composite bioink to recreate a bioengineered cartilage and characterized by label-free TPEF for cell autofluorescence and SHG microscopy for collagen fibrils formation	SHG and TPEF identified label-free vital cells within ECM. The addition of alginate to the bioink enhanced the iPSCs proliferation, differentiation (reduced expression of pluripotency markers) and the production of collagen-II
Syverud et al. (2017)	TPEF SHG	Primary murine muscle. cells	Structural and metabolic study of 3D skeletal engineered muscle tissue before implantation in the injured site of muscle loss. Collagen and myosin observed with SHG and redox ratio between NADH and FAD with TPEF. The engineered tissues were exposed to different culture conditions (metabolic stressed and steroid supplemented) to establish if changes in cell behavior were observable	Multiple time-points were observed to assess the metabolic activity of both monolayer and 3D engineered tissue to characterized differentiated and metabolically stressed tissues: an overall decrease of metabolic activity over time (0–8 min) in both sample type. Increase of metabolic activity during force production was not significant. Structural maturity assessed with SHG of myosin and collagen with sarcomere length and distribution. Correlation between myosin density and force production estimated both with SHG and force transducers
Costa Moura et al. (2018), Moura et al. (2019)	TPEF SHG CARS	Human fetal skeletal stem cells	Temporal assessment of cartilage tissue formation through stem cell differentiation and validation of bioengineered cartilage through label-free microscopy	Quantification of collagen fibers (width, length and straightness) within engineered cartilaginous tissue at different time points. The number and the size of the cells within the pellet was obtained from CARS to gain the amount of collagen fibers per cell. CARS and SHG were used to characterize collagen and its protein composition and resonance in the whole volume
Cong et al. (2019)	TPEF (FLIM)	Metastatic murine breast cancer cell line (4T1)	Metabolic assessment of tumor cells in 3D culture model made by collagen matrix exposed to two different drugs. Different levels of NAD(P)H lifetimes were found in between the flat and 3D culture configurations while exposed to drugs	Cell behavior, distribution and assembling depended on substrates stiffness and differed from 2D and 3D culture configuration. NAD(P)H levels and lifetime varied upon cell configuration resulting more longer in 3D systems. Metabolic results were obtained from the two culture configurations also under drug treatments to inhibit monocarboxylate transporters (pyruvate and lactate transport after glycolysis) to assess the reliability of FLIM measurements
Kaushik et al. (2019)	TPEF	Human embryonic-stem cell-derived endothelial cells and primary human pericytes	Non-invasive monitoring of autofluorescent signal of vessel formation in two dynamic bioreactors through recirculating and continuous media flows. Cells encapsulated in hydrogels self-assembled in 3D vascular networks stable in time	Fluorescence signal from NADH and NAD(P)H enabled to identify self-assembled vessels. Diameter, density, branch nodes amount and anisotropy of networks in static, recirculating, and continuous flow conditions after 6 days of study were estimated. Results from label-free TPEF were compared with immunocytochemistry and confocal fluorescence microscopy with similar trends in morphological parameters but different absolute values due to sample staining. Faster TPEF scanning systems will enable time dependent study in vessel formation
Okkelman et al. (2020)	TPEF (FLIM)	Intestinal stem cells Lgr5+	Metabolic study involving intestinal stem cells during development and differentiation toward 3D intestinal organoid via NAD(P)H lifetime fluorescence and TPEF for GFP	Organoid metabolism was established exposing stem cells (GFP) and differentiated cells (unlabeled) to different levels of glucose (low and high) and no pyruvate. TPEF-FLIM revealed any changes in lifetime in the three conditions for unlabeled cells while lower and variables lifetimes were obtained for stem cells thus suggesting a different metabolic trend. Data were confirmed by the fraction of protein bound. Stem cells exposed to low level of glucose showed an oxidative metabolism than differentiated cells
Sood et al. (2019)	TPEF-FLIM CARS	Primary human pediatric and adult brain tumor cells and human induced neural stem cells	Metabolic assessment of tumor cells in 3D silk protein scaffold embedded with ECM and hydrogels for engineered *in vitro* platform to establish the role of the material stiffness in the expression of different tumor subtypes. Further characterization of the model involved the use of CARS microscopy to quantify the lipid content and correlate the drug permeability	FLIM microscopy revealed variable tumor proliferation with different metabolic behavior and redox ratio trends depending on hydrogel compositions. A cross-check of lipid droplets formed during adult brain tumor cells was done by BODIPY fluorescence staining and chemically specific CARS in the C-H stretch region

## Conclusion

This first NLO imaging assessment is a starting point for tissue engineering, oncological research and drug testing applications since conventional methods commonly used to assess phenotypic expression are often disruptive (gene expression analyses) and perturbative (immuno-histochemical staining) thus limiting long-term studies. In this review we presented the main NLO techniques for functional observation of label-free and vital biological specimens, such as TPEF, SHG, THG, CARS, and SRS microscopy and their physical principles. Each of these methods exploits different properties of the light-matter interaction and it is useful to image characteristics of biological specimens in unlabeled conditions. TPEF is recognized as the main NLO techniques capable to excite endogenous molecules and co-factors, and exogenous fluorophores, reaching a great penetration depth (>500 μm). If combined with a fast detector such as a time-correlated single photon counter, it is possible to perform TPEF-FLIM for lifetime measurements of auto-fluorescent molecules, such as NADH and flavoproteins. SHG microscopy, instead, is very useful to reveal label-free signal from a non-centrosymmetric medium such as collagen fibrils, myosin and microtubules. THG, instead, enables the observation of interfaces within media with different refraction indexes, for example, enabling the identification of lipid-water interfaces in cellular vesicles. Vibrational-based CRS techniques excite the vibrational resonance frequency of the main chemical bonds with a specific and distinguishable fingerprint to investigate the chemical composition of the specimens. Finally, CARS and SRS allow the characterization of the biochemical composition of living samples both at the single-cell and at the tissue levels by measuring the vibrational spectrum of the specimen at every position, thus quantifying the concentration of e.g., DNA, lipids and proteins. Despite, all the advantages related to these techniques i.e., 3D sectioning capability, vital imaging and label-free acquisitions, they present a resolution close to the one achievable with confocal microscopy (lateral 200–300 nm and axial 600–900 nm). Currently, single-photon microscopy techniques are used for super resolution microscopy (SRM) such as stimulated emission depletion (STED) microscopy, thus enabling to image molecules with dimension under the diffraction limit (<100 nm) thanks to specific fluorophores or combinations of techniques to extrapolate signals. However, even if the development of SRM-NLO is recent, we believe that this combination results in efficient functional microscopy enabling the observation of dynamic processes at the molecular level in the absence of dyes. Multi-modal and multi-spectral nonlinear imaging will be of a great interest for tissue engineering applications thanks to the variety of biological information observable, from single cell components to heterogenous tissues, without the use of staining in unperturbed and non-destructive way. Hence, future challenging applications would be in clinic since the need for rapid and label-free diagnostic tools is far from being satisfied. In fact, pre-operatory assessments and surgical observations at the cellular level would highly reduce the risk of tissue damage due to unprecise excisions and uncertainty in the identification of injured/pathological sites.

## Author Contributions

VP wrote the manuscript and selected published scientific articles. EJ, DP, and MR structured the manuscript. EJ, RO, GC, DP, and MR reviewed the work. DP and MR provided financial support. All authors contributed to the article and approved the submitted version.

## Conflict of Interest

MR, GC, and RO were co-founders of a university spin-off company, MOAB S.r.l., and hold shares. The remaining authors declare that the research was conducted in the absence of any commercial or financial relationships that could be construed as a potential conflict of interest.

## Abbreviations

2D: bi-dimensional
3D: three-dimensional
Caco-2: Caucasian colon adenocarcinoma cells
CARS: coherent anti-Stokes Raman scattering
C-H: carbon-hydrogen
CRS: coherent Raman scattering
DNA: deoxyribonucleic acid
ECM: extracellular matrix
FAD: flavin-adenine dinucleotide
FLIM: fluorescent lifetime imaging
FP: flavoprotein
FRAP: fluorescence recovery after photobleaching
FRET: florescence resonance energy transfer
FTIR: Fourier transform infrared spectroscopy
GFP: green fluorescent protein
GTP: guanosine-5’-triphosphate
HEK: human embryonic kidney cells
HelaS3: Henrietta Lacks cervical cancer cells
iPSC: induced pluripotent stem cells
MCF: Michigan cancer foundation
MDA-MB: M. D. Anderson metastasis breast cancer
MSCs: mesenchymal stem cells
NAD(P)H: nicotinamide adenine dinucleotide phosphate
NLO: nonlinear optical
NRB: non-resonant background
PC: polycarbonate
PET: polyethylene
PTFE: polytetrafluoroethylene
RFP: red fluorescent protein
SEM: scanning electron microscopy
SHG: second harmonic generation
SR: spontaneous Raman
SRG: stimulated Raman gain
SRL: stimulated Raman loss
SRM: super resolution microscopy
SRS: stimulated Raman scattering
STED: stimulated emission depletion
THG: third harmonic generation
TPEF: two-photon excited fluorescence
YFP: yellow fluorescent protein.
